# Atopic dermatitis; to use or not to use the corticosteroids; mother's dilemma! A case report

**DOI:** 10.1186/2045-7022-5-S1-P9

**Published:** 2015-03-11

**Authors:** Erjola Piluri Ziu, Eris Mesonjesi, Irena Savo, Blerina Bregu, Maria Zoto

**Affiliations:** 1 American Hospital, Department of Allergy and Clinical Immunology, Tirana; Albania; 2 University Hospital Center "Mother Teresa", Department of Allergy and Clinical Immunology, Tirana; Albania; 3 American Hospital, Department of Dermatology, Tirana, Albania

## Background

Atopic dermatitis (AD) is a chronic, inflammatory skin disorder of the childhood. The patient with AD is affected by the social stigma of a visible skin condition. Topical corticosteroids being the first-line pharmacologic treatments can effectively control atopic flares in the majority of patients.

## Methods

We describe a case of severe AD untreated cause of mother's corticosteroid phobia.

## Case report

A 10-years-old female patient was presented with a poorly controlled AD. Widespread erythemas, lichenified plaques, were present on the face, neck, trunk and extremities. Numerous excoriations were seen. The patient complained of an unbearable pruritus. The patient had a history of mild AD since early infancy, managed by emollients. Allergic rhinitis was diagnosed at the age of 8. The patient had no family history for atopy. The last year the patient experienced an aggravation of her condition as presented. The little girl for one whole year was living with her pruritus, with the stigma and the numerous nick names sets by the other children. Our patient did not use topical steroids due to the girl's mother concerns about the corticosteroids side effects, despite the numerous prescriptions of allergist and dermatologists. SCORAD at first visit was 93.8. Laboratory tests showed levels of specific IgE for D. pteronyssinus, Timothy grass pollen and P judaica >100 kU/ml. She was not sensitized to food allergens. We did not perform skin tests cause of the severity of her AD. We made four consecutive appointments, in order to explain to the mother the nature of AD, the treatment needed, the side effects of the topical steroids, but we did not achieved results. She declare, that she is going to visit her child outside the country, where no one is going to prescribe to her little girl topical corticosteroids.

## Conclusion

AD is a chronic, inflammatory, itchy, episodic, skin condition that develops in early childhood in the majority of cases. Severe AD has the tendency to follow a persistent course and can be associated with a lack of self-confidence that can impair social development. Topical corticosteroids are gold standard for atopic dermatitis. The "corticosteroid phobia" is a phenomenon mentioned often in the literature. It is an irrational fear and anxiety of patients about using topical corticosteroid preparations. One of the most important sources of information about corticosteroids must be the doctor's consultation.

## Consent

Written informed consent was obtained from the patient for publication of this abstract and any accompanying images. A copy of the written consent is available for review by the Editor of this journal.

